# A survey of views about infant consciousness

**DOI:** 10.1093/nc/niag014

**Published:** 2026-06-01

**Authors:** Claudia Passos-Ferreira, David J Chalmers

**Affiliations:** Center for Bioethics, New York University, New York, NY, United States; Department of Philosophy, New York University, 5 Washington Place, New York, NY 10003, United States

**Keywords:** consciousness, infant consciousness, methodology, theories and models

## Abstract

We surveyed attendees at two leading conferences on consciousness, asking for their views on central issues concerning infant consciousness. These include questions about whether newborns are conscious, when consciousness and self-consciousness emerge, where in the brain the neural basis of infant consciousness is found, what sorts of consciousness infants may have, and how best to study infant consciousness, as well as questions about which creatures are conscious and which theories of consciousness are preferred. A majority of respondents favoured the view that newborns are conscious. Pluralities favored the view that consciousness emerges prenatally after 24 weeks of gestation and the view that self-consciousness emerges postnatally after 6 months of age. Large majorities favoured the views that infants have sensory and affective consciousness (with fewer favouring cognitive and agentive consciousness). A plurality favored the view that the neural basis of infant consciousness is in sensory cortex. Majorities favored behavioural and neural markers (but not theories of consciousness) as a guide to infant consciousness.

**Highlights of “A survey of views about infant consciousness”**

- A survey of neuroscientists, psychologists, philosophers, and others.
- A large majority favors the view that newborn infants are conscious.
- A plurality favors late pre-natal emergence of consciousness.
- A plurality favors late post-natal emergence of self-consciousness.
- Majorities favor newborns having sensory and affective consciousness, but not cognitive or agentive consciousness.

## Introduction

The study of consciousness in infants has been a recently active area in the science and philosophy of consciousness. Central questions in the area (as formulated by Passos-Ferreira, forthcoming, with inspiration from Güzeldere 1997 and Bayne et al. 2023, among others) include:

1. The whether question (existence): Are newborn infants conscious?
2. The when question (development): When does consciousness emerge in development?
3. The where question (neurobiology): What is the brain basis of infant consciousness?
4. The what question (phenomenology): What is it like to be an infant, and what sorts of consciousness do they have?
5. The how question (methodology): How can we study infant consciousness?

We set out to survey researchers and others interested in this area about their answers to these questions, along with a few connected questions about infants and consciousness.

Previous survey work has surveyed consciousness researchers’ views of consciousness (Francken et al. 2022) and academic philosophers’ views on philosophical questions including questions about consciousness (Bourget and Chalmers 2023). These two surveys both asked a single question about infant consciousness, but there has been no prior survey devoted to the topic that we know of.

Why is a survey of interest? First, it gives us a better sense of the sociology of this growing field. Second, it is common to appeal to expert opinion in discussing infant consciousness, and those discussions will be better grounded when we have a clearer understanding of what expert opinion is. Third, the relative popularity of views is often used to justify paying attention to some views while setting others aside, and again, this sort of justification will be better grounded with better knowledge about relative popularity.

This survey was a preliminary exercise in data collection and does not aim to test any scientific hypotheses. It is subject to many limitations that we will discuss, but we hope to prepare the ground for future work that goes beyond these limitations.

## Methods and materials

We surveyed attendees at two conferences in 2025: a one-off conference on infant consciousness, held at New York University (NYU) on 28 February and 1 March 2025, and the 28th annual meeting of the Association for the Scientific Study of Consciousness (ASSC), held in Heraklion, Crete, on 6–9 July 2025. This restriction allowed us to focus on well-controlled populations of mostly scientists and philosophers with an interest in our key topics. At the start of both conferences, we emailed all registered participants in the conference encouraging them to take the survey. We also announced the survey in relevant sessions at the conferences.

The study protocol was reviewed and approved by the New York University Human Research Protection Program Institutional Review Board (IRB-FY2025–9986). All participants provided informed consent electronically prior to answering the survey questions. Participation was voluntary, and respondents could withdraw at any time. The survey was administered through Google Forms.

Aside from introductory material including consent and validation questions and questions asked in order to comply with General Data Protection Regulations (Europe) and Personal Information Protection Law (China), the survey included three demographic questions, a preamble, and eight substantive questions.

The three demographic questions were:

1. D1: What is your main field? Answer options: Neuroscience, philosophy, psychology, other academic field, non-academic field.
2. D2: Have you previously written about infants or consciousness? Answer options: infants, consciousness (with checkboxes allowing participants to select both).
3. D3: What is your role at the [NYU/ASSC] conference? Answer options: Presenter, non-presenting participant, online attendee, not at conference.

The preamble to the substantive questions was as follows:

1. In the questions below, please indicate your opinion about the correct answer to the question. If you lean toward or accept an answer, please indicate that answer. Full belief or certainty are not required.
2. In this survey, ‘consciousness’ always means phenomenal consciousness, or subjective experience. A creature is conscious if there is something it is like to be that creature.

Of the eight substantive questions, the first six were versions of the ‘whether,’ ‘when,’ ‘where,’ ‘what,’ and ‘how’ questions about infant consciousness described above, including two versions of the ‘when’ question for consciousness and for self-consciousness. The other two are more general questions about consciousness, concerning who is conscious (a ‘who’ question) and which theories of consciousness are most promising (a ‘which’ or ‘why’ question).

1. Q1: Are newborn human babies typically conscious? Answer options: Yes, no, agnostic/other.
2. Q2: When does consciousness first develop in a typical human life cycle (with full-term gestation)? Answer options: Early pre-natal (before 24 weeks of gestation), later pre-natal (around or after 24 weeks of gestation), at birth, early post-natal (before 6 months old), later post-natal (around or after 6 months old), agnostic/other.
3. Q3: When does self-consciousness first develop in a typical human life cycle.
4. (with full-term gestation)? Answer options: As for Q2.
5. Q4: What is the main neural basis of infant consciousness? Answer options (multiple responses allowed): Sensory cortex, frontal cortex, subcortical systems, agnostic/other.
6. Q5: What sorts of conscious experience does a newborn baby have? Answer options (multiple responses allowed): Sensory, affective, agentive, cognitive, agnostic/other.
7. Q6: Which of these provides the best guide to infant consciousness? Answer options (multiple responses allowed): Behavioral markers, neural markers, theories of consciousness, agnostic/other.
8. Q7: For which of these groups are some members conscious? Answer options (multiple responses allowed): Adult humans, cats, fish, flies, worms, plants, particles, current AI systems, future AI systems.
9. Q8: Which of these theories of consciousness do you find most promising? Answer options (multiple responses allowed): Attention schema theory, biological theories, computational theories, dualist theories, functionalist theories, global neuronal workspace theory, higher-order theories, idealist theories, illusionist theories, integrated information theory, panpsychist theories, predictive processing theories, quantum theories, recurrent processing theories, representational theories, agnostic/other.

## Results

A total of 247 people responded to the survey. At NYU, 94 people responded to the survey (out of 360 registered conference participants). At ASSC, 153 people responded to the survey (out of 775 registered conference participants).

### Demographics

**Figure 1:**
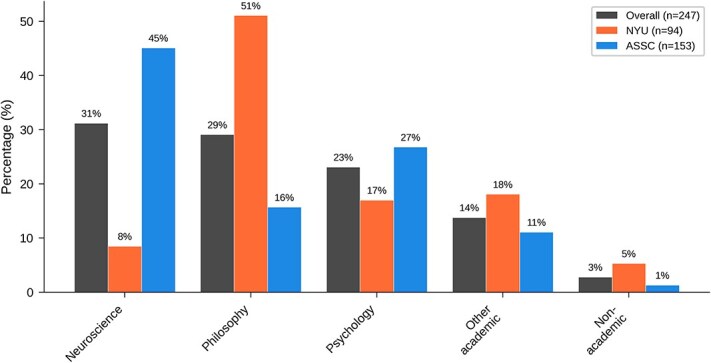
Graphical results of demographic question 1: What is your main field?

Results for the demographic questions were as follows:

Table 1 and Figure 1 show results for ‘What is your main field’. The best-represented fields overall are neuroscience and philosophy, followed by psychology. The largest group at NYU was philosophy (51%, compared to 16% at ASSC), while the largest group at the ASSC conference was neuroscience (45%, compared to 8% at NYU). Psychology was somewhat better represented at ASSC (27%, compared to 18% at NYU).

**Table 1:** Results of demographic question 1: What is your main field?

Field	Overall (n = 247)	NYU (n = 94)	ASSC (n = 153)
Neuroscience	77 (31.2%)	8 (8.5%)	69 (45.1%)
Philosophy	72 (29.1%)	48 (51.1%)	24 (15.7%)
Psychology	57 (23.1%)	16 (17.0%)	41 (26.8%)
Other academic field	34 (13.8%)	17 (18.1%)	17 (11.1%)
Non-academic field	7 (2.8%)	5 (5.3%)	2 (1.3%)

These numbers may reflect the fact that while both conferences were interdisciplinary, the NYU conference was organized and attended especially by philosophers, while the ASSC 28 conference was organized and attended especially by cognitive neuroscientists. In a larger demographic survey at the ASSC 28 conference conducted by ASSC organizers (with 477 out of 775 attendees responding), 43% of respondents were neuroscientists, 24% were psychologists, 11% were philosophers, 6% were computer scientists, and 4% were clinicians.

**Figure 2:**
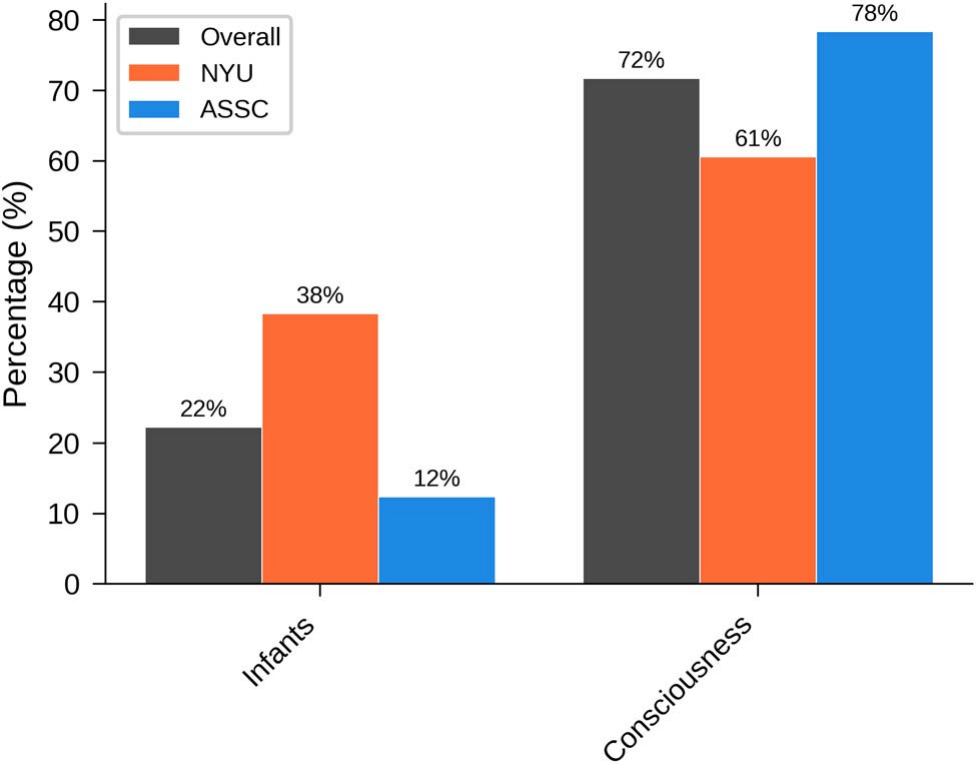
Graphical results of demographic question 2: Have you previously written about infants or consciousness?

Table 2 and Figure 2 show results for ‘Have you previously written about infants or consciousness’. Thirty-four respondents (14%) checked both boxes, while 49 (20%) checked neither. A background in consciousness issues was much more common overall. Unsurprisingly, given the topics of the conferences, a background in infant issues was more common at the NYU conference than at the ASSC conference, while a background in consciousness issues was somewhat more common at the ASSC conference than at the NYU conference.

**Table 2:** Results of demographic question 2**:** Have you previously written about infants or consciousness?

Option checked	Overall (n = 247)	NYU (n = 94)	ASSC (n = 153)
Infants	55 (22.3%)	36 (38.3%)	19 (12.4%)
Consciousness	177 (71.7%)	57 (60.6%)	120 (78.4%)

**Figure 3:**
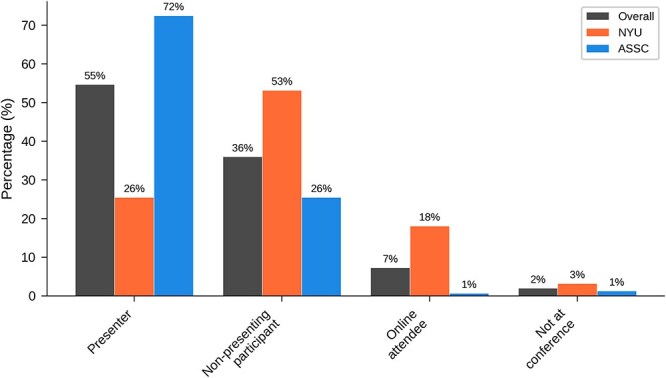
Graphical results of demographic question 3: What is your role at the conference?

Table 3 and Figure 3 show results for ‘What is your role at the conference?’. Overall, just over half of respondents were conference presenters (including keynote speakers, symposium participants, and poster presenters at both conferences, and concurrent talk presenters at ASSC). A higher proportion of ASSC respondents were presenters (73% versus 26% at NYU), reflecting the relative numbers of presenters at the two conferences.

**Table 3:** Results of demographic question 3: What is your role at the conference?

Role	Overall (n = 247)	NYU (n = 94)	ASSC (n = 153)
Presenter	135 (54.7%)	24 (25.5%)	111 (72.5%)
Non-presenting participant	89 (36.0%)	50 (53.2%)	39 (25.5%)
Online attendee	18 (7.3%)	17 (18.1%)	1 (0.7%)
Not at conference/ Other	5 (2.0%)	3 (3.2%)	2 (1.3%)

We also encouraged (but did not require) participants to include their email addresses for validation purposes. Around 80% of participants reported an email address. These addresses indicated one repeat respondent (both responses at NYU), for which we kept the first responses and discarded the second. We examine potential differences between the anonymous and non-anonymous groups in the discussion section.

### Substantive questions

**Figure 4:**
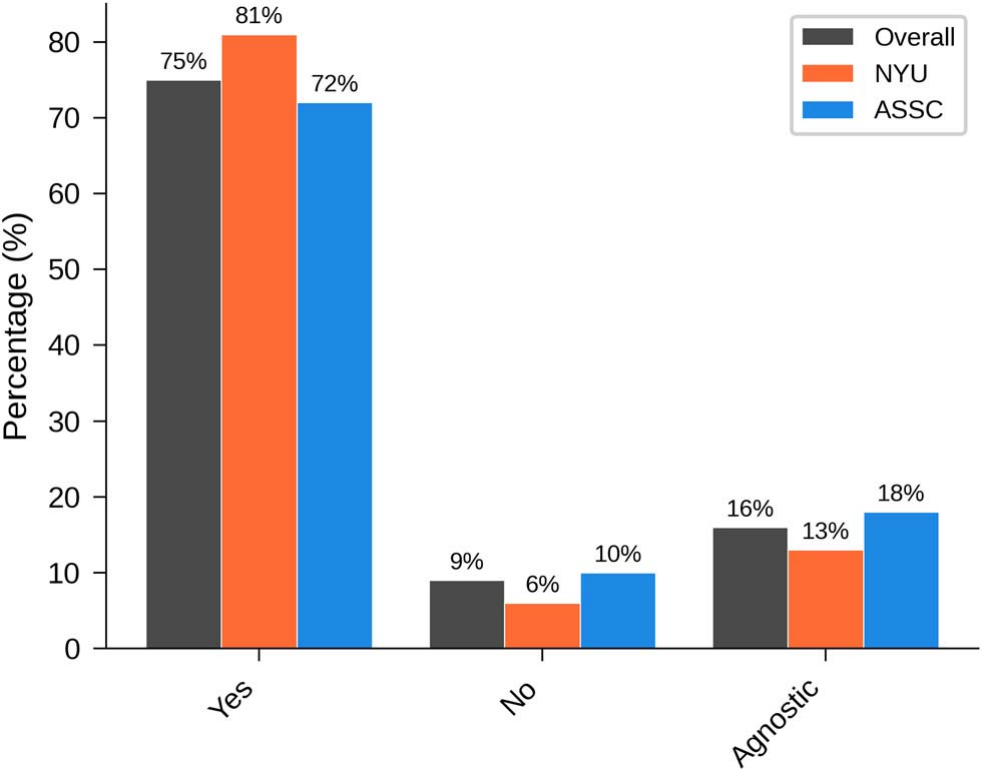
Graphical results of main question 1: Are newborn human babies typically conscious?

Table 4 and Figure 4 show overall results for ‘Are newborn human babies typically conscious?’ Across both groups, a substantial majority favored the view that newborns are typically conscious.

**Table 4:** Overall results of main question 1**:** Are newborn human babies typically conscious?

Answer	All	NYU	ASSC
Yes	186 (75%)	76 (81%)	110 (72%)
No	21 (9%)	6 (6%)	15 (10%)
Agnostic	40 (16%)	12 (13%)	28 (18%)

**Figure 5:**
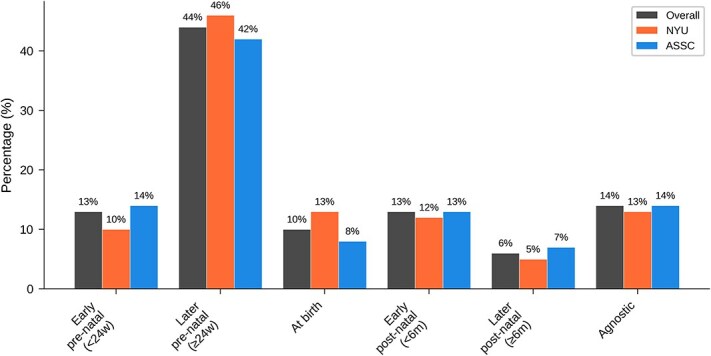
Graphical results of main question 2: When does consciousness first develop in a typical human life cycle?

Table 5 and Figure 5 show overall results for ‘When does consciousness first develop in a typical human life cycle?’ A strong plurality (that is, the largest subgroup but not an absolute majority) in all groups favoured the late pre-natal view (around or after 24 weeks of gestation; note that weeks of gestation are standardly understood as weeks since last menstrual period), with smaller numbers favoring early pre-natal and early post-natal views.

**Table 5:** Overall results of main question 2: When does consciousness first develop in a typical human life cycle?

Answer	All	NYU	ASSC
Early pre-natal (< 24w gestation)	32 (13%)	10 (10%)	22 (14%)
Later pre-natal (> 24w gestation)	108 (44%)	43 (46%)	65 (42%)
At birth	24 (10%)	12 (13%)	12 (8%)
Early post-natal (< 6 m old)	32 (13%)	12 (12%)	20 (13%)
Later post-natal (> 6 m old)	16 (6%)	5 (5%)	11 (7%)
Agnostic	34 (14%)	12 (13%)	22 (14%)

**Figure 6:**
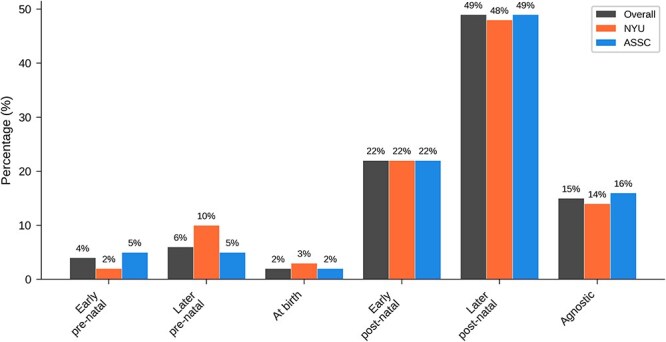
Graphical results for main question 3: When does self-consciousness first develop in a typical human life cycle?

Table 6 and Figure 6 show overall results for ‘Are newborn human babies typically self-conscious?’ Where a plurality of respondents to the previous question favoured a late pre-natal view of the emergence of consciousness, around half of respondents to this question favored a late *post*-natal view (after 6 months old) of the emergence of *self*-consciousness. Around 20% favoured an early post-natal view, and smaller numbers favoured other options.

**Figure 7:**
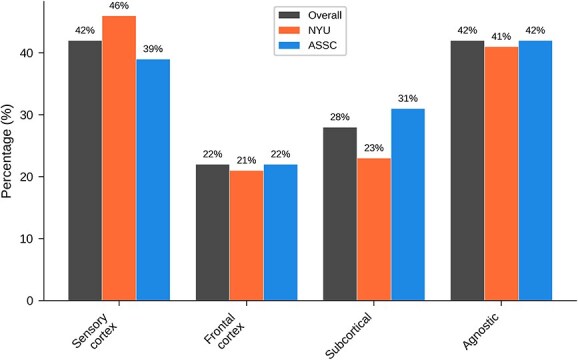
Graphical results for main question 4: What is the main neural basis of infant consciousness?

Table 7 and Figure 7 show overall results for ‘What is the main neural basis of infant consciousness?’ Here a plurality favoured sensory cortex (with an equally high number of agnostics), followed by subcortical systems and frontal cortex.

**Figure 8:**
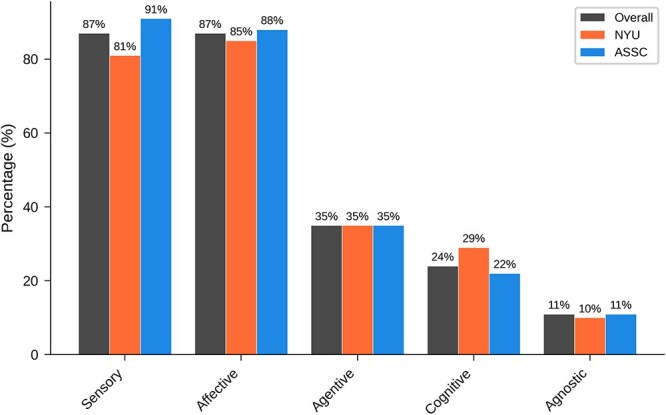
Graphical results for main question 5: What sorts of conscious experience does a newborn baby have?

Table 8 and Figure 8 show overall results for ‘What sorts of conscious experience does a newborn baby have?’ Here a large majority of respondents favored the view that newborns have sensory and affective consciousness, with minorities favoring agentive and cognitive consciousness.

**Table 6:** Overall results for main question 3: When does self-consciousness first develop in a typical human life cycle?

Answer	All	NYU	ASSC
Early pre-natal	10 (4%)	2 (2%)	8 (5%)
Later pre-natal	16 (6%)	9 (10%)	7 (5%)
At birth	6 (2%)	3 (3%)	3 (2%)
Early post-natal	55 (22%)	21 (22%)	34 (22%)
Later post-natal	120 (49%)	45 (48%)	75 (49%)
Agnostic	38 (15%)	13 (14%)	25 (16%)

**Table 7:** Overall results for main question 4**:** What is the main neural basis of infant consciousness?

Answer	All	NYU	ASSC
Sensory cortex	103 (42%)	43 (46%)	60 (39%)
Frontal cortex	54 (22%)	20 (21%)	34 (22%)
Subcortical	69 (28%)	22 (23%)	47 (31%)
Agnostic	103 (42%)	39 (41%)	64 (42%)

**Table 8:** Overall results for main question 5: What sorts of conscious experience does a newborn baby have?

Answer	All	NYU	ASSC
Sensory	215 (87%)	76 (81%)	139 (91%)
Affective	215 (87%)	80 (85%)	135 (88%)
Agentive	87 (35%)	33 (35%)	54 (35%)
Cognitive	60 (24%)	27 (29%)	33 (22%)
Agnostic	26 (11%)	9 (10%)	17 (11%)

**Figure 9:**
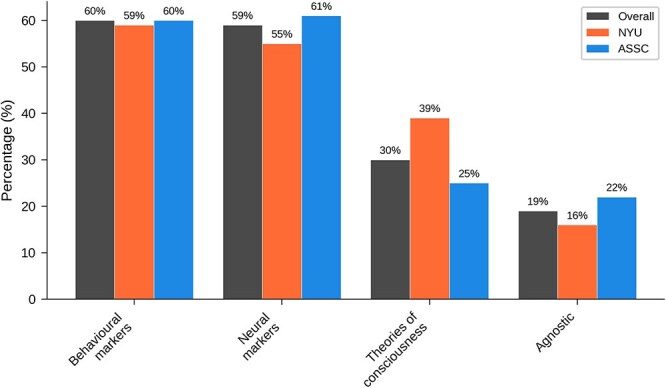
Graphical results for main question 6: Which of these provides the best guide to infant consciousness?

Table 9 and Figure 9 show overall results for ‘Which of these provides the best guide to infant consciousness?’ Here a majority favoured behavioural and neural markers, with a minority favoring theories of consciousness.

**Table 9:** Overall results for main question 6: Which of these provides the best guide to infant consciousness?

Answer	All	NYU	ASSC
Behavioral markers	147 (60%)	55 (59%)	92 (60%)
Neural markers	146 (59%)	52 (55%)	94 (61%)
Theories of consciousness	75 (30%)	37 (39%)	38 (25%)
Agnostic	48 (19%)	15 (16%)	33 (22%)

**Figure 10:**
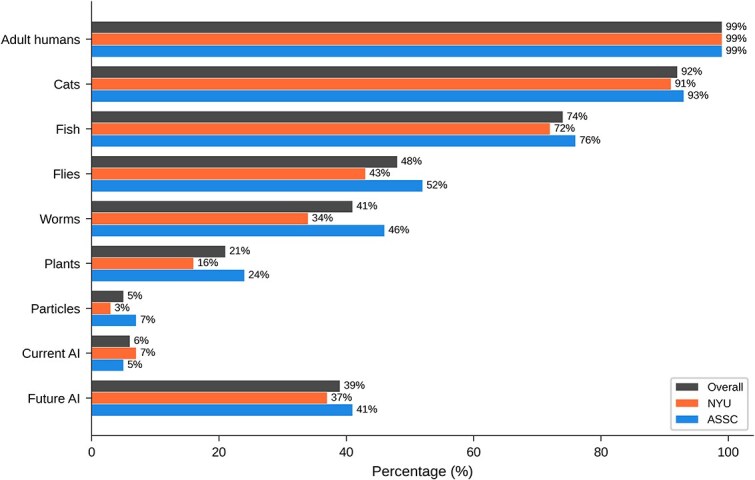
Graphical results for main question 7: For which of these groups are some members conscious?

Table 10 and Figure 10 show overall results for ‘For which of these groups are some members conscious?’ Almost everyone favoured consciousness in humans and, to a slightly lesser extent, in cats. A substantial majority favoured consciousness in fish, while around half favored consciousness in flies. Strikingly, 20% favoured consciousness in some plants. Few respondents held that current AI systems are conscious, while a substantial minority held that future AI systems will be conscious. Note that there was no ‘agnostic’ option for this question.

**Table 10:** Overall results for main question 7: For which of these groups are some members conscious?

Answer	All	NYU	ASSC
Adult humans	245 (99%)	93 (99%)	152 (99%)
Cats	228 (92%)	86 (91%)	142 (93%)
Fish	184 (74%)	68 (72%)	116 (76%)
Flies	119 (48%)	40 (43%)	79 (52%)
Worms	102 (41%)	32 (34%)	70 (46%)
Plants	51 (21%)	15 (16%)	36 (24%)
Particles	13 (5%)	3 (3%)	10 (7%)
Current AI	14 (6%)	7 (7%)	7 (5%)
Future AI	97 (39%)	35 (37%)	62 (41%)

**Figure 11:**
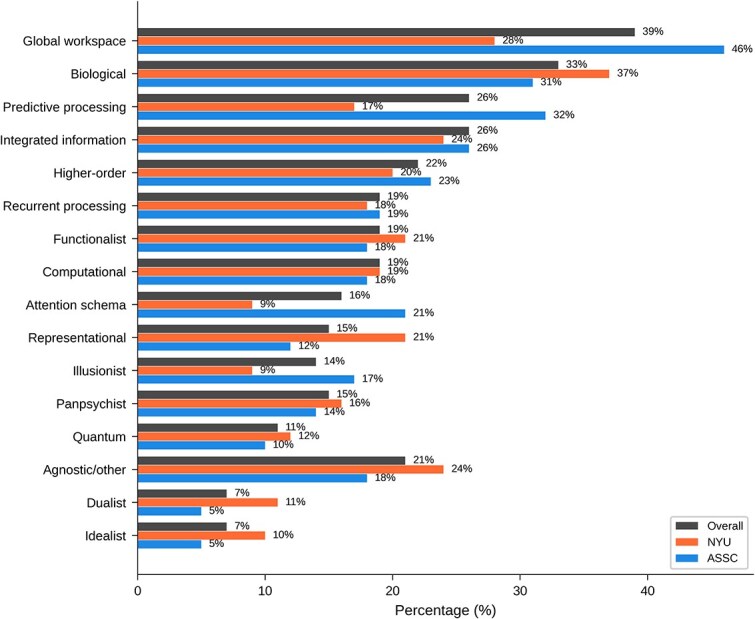
Graphical results for main question 8: Which of these theories of consciousness do you find most promising?

Table 11 and Figure 11 show overall results for ‘Which of these theories do you find most promising?’ The global neuronal workspace theory attracted the most support overall, followed by biological theories, predictive processing theories, integrated information theory, and higher-order theories. It is notable that in the NYU group, biological theories were the most popular, while at ASSC, both global workspace and predictive processing theories were more popular than biological theories.

**Table 11:** Overall results for main question 8: Which of these theories of consciousness do you find most promising?

Answer	All	NYU	ASSC
Global workspace	96 (39%)	26 (28%)	70 (46%)
Biological	82 (33%)	35 (37%)	47 (31%)
Predictive processing	65 (26%)	16 (17%)	49 (32%)
Integrated information	63 (26%)	23 (24%)	40 (26%)
Higher-order	54 (22%)	19 (20%)	35 (23%)
Recurrent processing	46 (19%)	17 (18%)	29 (19%)
Functionalist	48 (19%)	20 (21%)	28 (18%)
Computational	46 (19%)	18 (19%)	28 (18%)
Attention schema	40 (16%)	8 (9%)	32 (21%)
Representational	38 (15%)	20 (21%)	18 (12%)
Illusionist	34 (14%)	8 (9%)	26 (17%)
Panpsychist	36 (15%)	15 (16%)	21 (14%)
Quantum	26 (11%)	11 (12%)	15 (10%)
Agnostic/Other	51 (21%)	23 (24%)	28 (18%)
Dualist	18 (7%)	10 (11%)	8 (5%)
Idealist	17 (7%)	9 (10%)	8 (5%)

We were not testing hypotheses in this survey, but it may be of descriptive interest to see whether NYU-ASSC differences are significant. After correcting for multiple comparisons (Holm-Bonferroni correction for 41 NYU-ASSC comparisons in Q1-Q8), there are no significant NYU-ASSC differences (*P* < .05) on answers in Q1-Q8. Before correction, there are significant differences only on Q5, Q6, and Q8. On Q5, significantly more ASSC respondents (*P* < .05) favored newborn babies having sensory experience. On Q6, significantly more NYU respondents favoured theories of consciousness as a method. On Q8, significantly more ASSC respondents favored global workspace theory (*P* < .01), predictive processing theories (*P* < 0.05), and attention schema theory (*P* < .05). These differences remain significant even after controlling for field and expertise.

### Breakdown by field and expertise

In what follows, we break down responses to Q1-Q8 by field (neuroscience, philosophy, psychology), and by expertise (infants, consciousness, and infant consciousness).

Table 12 shows results broken down by field and expertise for ‘Are newborn babies typically conscious?’. More philosophers and psychologists than neuroscientists favoured the view that newborns are typically conscious.

**Table 12:** Results by field and expertise for main question 1: Are newborn babies typically conscious?

Answer	All	Neuro	Phil	Psych	Infant	Consc	Infant+Consc
Yes	186 (75%)	51 (66%)	60 (83%)	45 (79%)	44 (80%)	134 (76%)	27 (79%)
No	21 (9%)	9 (12%)	5 (7%)	2 (4%)	4 (7%)	16 (9%)	4 (12%)
Agnostic	40 (16%)	17 (22%)	7 (10%)	10 (18%)	7 (13%)	27 (15%)	3 (9%)

Table 13 shows results broken down by field and expertise for ‘When does consciousness first develop in a typical human life cycle?’ Somewhat fewer neuroscientists favor the later pre-natal view. More infant specialists favor the ‘at birth’ view.

**Table 13:** Results by field and expertise for main question 2: When does consciousness first develop in a typical human life cycle?

Answer	All	Neuro	Phil	Psych	Infant	Consc	Infant+ Consc
Early pre-natal	32 (13%)	9 (12%)	7 (10%)	5 (9%)	3 (5%)	24 (14%)	3 (9%)
Later pre-natal	108 (44%)	29 (38%)	38 (53%)	25 (44%)	25 (45%)	78 (44%)	15 (44%)
At birth	24 (10%)	10 (13%)	5 (7%)	6 (11%)	10 (18%)	13 (7%)	5 (15%)
Early post-natal	32 (13%)	11 (14%)	6 (8%)	10 (18%)	10 (18%)	23 (13%)	6 (18%)
Later post-natal	16 (6%)	7 (9%)	4 (6%)	2 (4%)	3 (5%)	13 (7%)	2 (6%)
Agnostic	34 (14%)	11 (14%)	12 (17%)	8 (14%)	4 (7%)	25 (14%)	3 (9%)

Table 14 shows results broken down by field and expertise for ‘When does self-consciousness first develop in a typical human life cycle?’ Neuroscientists favored the early post-natal view somewhat more often and the late post-natal view somewhat less often than philosophers and psychologists.

**Table 14:** Results by field and expertise for main question 3: When does self-consciousness first develop in a typical human life cycle?

Answer	All	Neuro	Phil	Psych	Infant	Consc	Infant+ Consc
Early pre-natal	10 (4%)	5 (6%)	1 (1%)	1 (2%)	3 (5%)	7 (4%)	3 (9%)
Later pre-natal	16 (6%)	4 (5%)	4 (6%)	4 (7%)	5 (9%)	9 (5%)	3 (9%)
At birth	6 (2%)	2 (3%)	0 (0%)	2 (4%)	3 (5%)	5 (3%)	2 (6%)
Early post-natal	55 (22%)	22 (29%)	13 (18%)	12 (21%)	11 (20%)	35 (20%)	6 (18%)
Later post-natal	120 (49%)	33 (43%)	37 (51%)	31 (54%)	29 (53%)	90 (51%)	16 (47%)
Agnostic	38 (15%)	11 (14%)	15 (21%)	7 (12%)	3 (5%)	29 (16%)	3 (9%)

Table 15 shows results broken down by field and expertise for ‘What is the main neural basis of infant consciousness?’ Neuroscientists favored frontal cortex somewhat more often and sensory cortex somewhat less often than philosophers and psychologists.

**Table 15:** Results by field and expertise for main question 4: What is the main neural basis of infant consciousness?

Option	All	Neuro	Phil	Psych	Infant	Consc	Infant+ Consc
Sensory cortex	103 (42%)	28 (36%)	28 (39%)	29 (51%)	25 (45%)	76 (43%)	16 (47%)
Frontal cortex	54 (22%)	21 (27%)	14 (19%)	12 (21%)	10 (18%)	42 (24%)	7 (21%)
Subcortical	69 (28%)	21 (27%)	19 (26%)	16 (28%)	15 (27%)	52 (29%)	8 (24%)
Agnostic	103 (42%)	36 (47%)	31 (43%)	18 (32%)	22 (40%)	71 (40%)	14 (41%)

Table 16 shows results broken down by field and expertise for ‘What sorts of consciousness does a newborn baby have?’ Large majorities held that newborns have sensory and affective consciousness across all expertise groups, while fewer respondents attributed agentive and cognitive consciousness. Philosophers were slightly less favorable to sensory consciousness while being slightly more favorable to affective consciousness than other groups. Infant specialists were more likely than others to attribute all forms of consciousness to newborns.

**Table 16:** Results by field and expertise for main question 5: What sorts of consciousness does a newborn baby have?

Type	All	Neuro	Phil	Psych	Infant	Consc	Infant+ Consc
Sensory	215 (87%)	69 (90%)	58 (81%)	52 (91%)	51 (93%)	158 (89%)	33 (97%)
Affective	215 (87%)	67 (87%)	64 (89%)	51 (89%)	51 (93%)	158 (89%)	32 (94%)
Agentive	87 (35%)	26 (34%)	27 (38%)	15 (26%)	25 (45%)	55 (31%)	15 (44%)
Cognitive	60 (24%)	19 (25%)	17 (24%)	11 (19%)	19 (35%)	42 (24%)	12 (35%)
Agnostic	26 (11%)	11 (14%)	6 (8%)	3 (5%)	4 (7%)	19 (11%)	4 (12%)

Table 17 shows results broken down by field and expertise for ‘Which of these is the best guide to infant consciousness?’ Perhaps unsurprisingly, neuroscientists tended to favour neural markers, while psychologists (and philosophers) tended to favour behavioural markers.

**Table 17:** Results by field and expertise for main question 6: Which of these is the best guide to infant consciousness?

Guide	All	Neuro	Phil	Psych	Infant	Consc	Infant+ Consc
Neural	146 (59%)	52 (68%)	36 (50%)	35 (61%)	37 (67%)	107 (60%)	26 (76%)
Behavioral	147 (60%)	44 (57%)	41 (57%)	41 (72%)	39 (71%)	104 (59%)	23 (68%)
Theories	75 (30%)	19 (25%)	23 (32%)	20 (35%)	17 (31%)	50 (28%)	12 (35%)
Agnostic	48 (19%)	16 (21%)	8 (11%)	10 (18%)	7 (13%)	33 (19%)	5 (15%)

Table 18 shows results broken down by field and expertise for ‘Of which groups are some members conscious?’ Philosophers were somewhat less willing to extend consciousness to flies and worms, but more willing to extend consciousness to future AI systems, than were neuroscientists and psychologists. The worm and fly differences are significant before, but not after correcting for multiple comparisons.

**Table 18:** Results by field and expertise for main question 7: Of which groups are some members conscious?

Entity	All	Neuro	Phil	Psych	Infant	Consc	Infant+ Consc
Humans	245 (99%)	76 (99%)	72 (100%)	57 (100%)	54 (98%)	177 (100%)	34 (100%)
Cats	228 (92%)	69 (90%)	68 (94%)	52 (91%)	51 (93%)	165 (93%)	32 (94%)
Fish	184 (74%)	52 (68%)	58 (81%)	43 (75%)	38 (69%)	130 (73%)	23 (68%)
Flies	119 (48%)	35 (45%)	33 (46%)	28 (49%)	18 (33%)	86 (49%)	13 (38%)
Worms	102 (41%)	31 (40%)	23 (32%)	25 (44%)	12 (22%)	72 (41%)	9 (26%)
Plants	51 (21%)	17 (22%)	8 (11%)	12 (21%)	7 (13%)	35 (20%)	5 (15%)
Particles	13 (5%)	3 (4%)	2 (3%)	4 (7%)	1 (2%)	9 (5%)	1 (3%)
Current AI	14 (6%)	3 (4%)	5 (7%)	4 (7%)	3 (5%)	10 (6%)	3 (9%)
Future AI	97 (39%)	29 (38%)	31 (43%)	19 (33%)	21 (38%)	78 (44%)	17 (50%)

Table 19 shows results broken down by field and expertise for ‘Which of these theories of consciousness do you find most promising?’ The global neuronal workspace theory, predictive processing, higher-order, and attention schema views were disproportionately preferred by psychologists. The integrated information view and agnosticism were disproportionately favored by neuroscientists. Biological, functionalist, idealist, and illusionist views were disproportionately favored by philosophers.

**Table 19:** Results by field and expertise for main question 8: Which of these theories of consciousness do you find most promising?

Theory	All	Neuro	Phil	Psych	Infants	Consc	Infant+ Consc
Global workspace	96 (39%)	31 (40%)	23 (32%)	26 (46%)	22 (40%)	72 (41%)	13 (38%)
Biological	82 (33%)	21 (27%)	27 (38%)	17 (30%)	18 (33%)	60 (34%)	12 (35%)
Predictive processing	65 (26%)	22 (29%)	15 (21%)	18 (32%)	13 (24%)	48 (27%)	9 (26%)
Integrated information	63 (26%)	22 (29%)	11 (15%)	14 (25%)	21 (38%)	47 (27%)	12 (35%)
Higher-order	54 (22%)	14 (18%)	15 (21%)	15 (26%)	10 (18%)	44 (25%)	8 (24%)
Recurrent-proc.	46 (19%)	13 (17%)	15 (21%)	12 (21%)	11 (20%)	37 (21%)	7 (21%)
Functionalist	48 (19%)	8 (10%)	22 (31%)	10 (18%)	12 (22%)	36 (20%)	7 (21%)
Computational	46 (19%)	13 (17%)	12 (17%)	10 (18%)	11 (20%)	32 (18%)	8 (24%)
Attention schema	40 (16%)	12 (16%)	6 (8%)	14 (25%)	10 (18%)	32 (18%)	8 (24%)
Representational	38 (15%)	5 (6%)	16 (22%)	13 (23%)	10 (18%)	33 (19%)	10 (29%)
Illusionist	34 (14%)	9 (12%)	12 (17%)	6 (11%)	6 (11%)	28 (16%)	5 (15%)
Panpsychist	36 (15%)	11 (14%)	10 (14%)	7 (12%)	5 (9%)	28 (16%)	4 (12%)
Quantum	26 (11%)	4 (5%)	7 (10%)	8 (14%)	3 (5%)	16 (9%)	2 (6%)
Agnostic/ Other	51 (21%)	19 (25%)	14 (19%)	6 (11%)	11 (20%)	35 (20%)	6 (18%)
Dualist	18 (7%)	3 (4%)	4 (6%)	3 (5%)	4 (7%)	11 (6%)	1 (3%)
Idealist	17 (7%)	2 (3%)	8 (11%)	4 (7%)	3 (5%)	13 (7%)	1 (3%)

As before, significance tests may be of descriptive interest, although they should be interpreted with caution as we were not testing hypotheses and participant numbers were small. When running an omnibus association test for effects of field or expertise on results of Q1-Q8, the only significant association found (before correcting for multiple comparisons) was between field and the results of Q8. When running an option-level test for effects of field or expertise on each specific answer to Q1-Q8, there were significant effects (before correcting for multiple comparisons) of field on three answers to Q8 (representational theory, functional theory, attention schema), and significant effects of expertise on some answers to Q5 (agentive experience), Q7 (worms, flies), and Q8 (representational theories, integrated information theory). After Holm-Bonferroni corrections for multiple comparisons, none of these effects were significant.

## Discussion

Some of the more notable results of the survey include (Q1) a large majority favored newborn consciousness, (Q2) a plurality (largest subgroup but not a majority) favored late pre-natal emergence of consciousness, (Q3) a plurality favored late-post-natal emergence of self-consciousness, (Q4) a plurality favoured sensory cortex as the basis of infant consciousness, (Q5) large majorities favoured infants having sensory and affective consciousness (but not cognitive or agentive consciousness), and (Q6) majorities favored behavioral and neural markers (but not theories of consciousness) as a guide to infant consciousness.

Approximately 20% of respondents chose to answer anonymously rather than provide an email address. It was possible in principle for anonymous respondents to influence the results by voting multiple times. To assess this possibility, we tested for any differences between anonymous respondents and non-anonymous respondents. Only one significant difference (*P* < .05 after correcting for multiple comparisons) was found: anonymous respondents were significantly less likely to check ‘agnostic/other’ on Q8 about the most promising theories of consciousness.

This survey does not give direct information about how views of infant consciousness change over time. However, by combining it with past surveys, we can get some information of this sort. The two most relevant prior surveys are the 2018–19 survey of ASSC participants’ views about consciousness (Francken et al. 2022) and the 2020 PhilPapers Survey of academic philosophers’ views on philosophical questions (Bourget and Chalmers 2023). There are a few points of overlap between our survey and these prior surveys where comparisons are meaningful. To maximize comparability, we restrict the current survey results to philosophers (when comparing to the 2020 PhilPapers survey) and to ASSC respondents (when comparing to the 2018/2019 ASSC survey).

*Are newborns conscious?* The 2020 PhilPapers survey asked a version of this question. 83% of respondents answered positively, as did the same percentage (83%) of philosophers in the current survey. The 2018–2019 ASSC survey asked whether babies have consciousness. 80% answered positively, compared to 72% of ASSC respondents in the current survey. The difference between ‘baby’ and ‘newborn’ may be responsible for some of the differences here.

*Which entities are conscious?* The 2020 PhilPapers survey asked almost exactly the same question asked here as Q7, with the following results. Adults 95% (versus 100% here), cats 89% (versus 95%), fish 65% (versus 81%), flies 35% (versus 45%), worms 24% (versus 32%), plants 7% (versus 11%), particles 2% (versus 3%), current AI systems 3% (versus 7%), future AI systems 39% (versus 44%). On all nine questions, somewhat more philosophers on the 2025 survey answered positively than on the 2020 survey. This may reflect the passage of time from 2020 to 2025, or a difference in the subgroups of philosophers responding to the surveys.

2018/2019 ASSC respondents were asked a similar question about consciousness across a slightly different group of entities: yourself (98% positive), other people (99%), monkey (98%), dog (91%), bat (77%), fish (60%), worm (26%), amoeba (14%), tree (8%), rock (3%), thermostat (1%), and current or future machines (67%). These compare to 2025 ASSC respondents: adult humans (99%), cats (93%), fish (76%), flies (52%), worms (46%), plants (24%), particles (7%), current AI (5%), and future AI (41%). On the clearest points of comparison (fish and worms), the 2025 numbers are higher. On other natural comparisons (humans-other people, cats-dogs, trees-plants, and future AI systems), the 2025 responses are more positive than 2018/2019 responses in all cases except humans (roughly the same) and future AI systems (lower in 2025). These results tend to suggest a trend toward more inclusive views of consciousness among ASSC participants.

*Which theories of consciousness are most promising?* Here the comparison points include predictive processing (58% positive in 2018/2019 versus 32% positive in 2025), global neuronal workspace (55% versus 46%), higher-order theories (51% versus 23%), local recurrency/recurrent processing (35% versus 19%), integrated information theory (43% versus 26%), and quantum theories (9% versus 10%). For most theories there is a drop from 2018/2019 to 2025 (possibly because of survey format), with a somewhat smaller drop for global workspace and quantum theories (though quantum theories had much lower figures overall).

## Limitations

These preliminary survey results are just a beginning in understanding the distribution of views about infant consciousness. The results have many limitations. We hope that future survey work will address all of these limitations.

First, we did not define key terms used in the survey questions, with the exception of ‘consciousness’ and ‘conscious’ which were defined in the preamble. We did not give definitions for key terms such as ‘self-consciousness,’ ‘affective,’ ‘gestation,’ ‘typical,’ ‘markers,’ and more. We also did not provide definitions of the central claims made by key theories of consciousness. This was a deliberate choice, as definitions themselves are controversial and often require further definitions in turn, but a downside is that various respondents may have understood key terms differently. Future surveys might aim to ask more precise questions using explicit definitions.

Second, we studied a limited population. These results are mostly limited to a relatively small group of conference-attending Western academic neuroscientists, philosophers, and psychologists who self-selected to participate in the survey. It cannot be assumed that the survey results will generalize to broader populations. A more inclusive survey would also gather data from other areas of the world and from other professions (especially from clinicians). It would also be valuable to have information about the distribution of views among the general public.

Third, the eight main survey questions were drawn from a limited range of topics and traditions. In future surveys, it would be especially useful to ask about ethical, medical, and social issues regarding infants and fetuses and to correlate these views with views about infant consciousness. It would be desirable to ask questions about many further issues, such as the status of premature infants, the role of birth, the role of the body, and the development of many different cognitive capacities. It would also be valuable to ask questions drawn from different traditions that engage with infant consciousness, including feminist traditions, global traditions, and phenomenological traditions, as well as multiple clinical and developmental traditions.

## Conclusion

Theorists writing about consciousness often make claims about the sociology of views about consciousness, their relative popularity, and where consensus lies. Theorists writing specifically about infant consciousness often do the same. For example, in an important recent article, (Bayne et al. 2023) write.

“There is, however, no consensus as to when consciousness first emerges and the range of candidate answers offered here is extremely wide. At one end of the spectrum are accounts that suggest that consciousness might be in place from as early as 24 to 26 weeks of gestational age, which is when thalamocortical connectivity is first established. At the other end of the spectrum are accounts according to which consciousness is unlikely to be in place significantly prior to the child’s first birthday.

This sociological discussion conveys a sense that late pre-natal views (after 24 weeks of gestation) and late post-natal views (after 6 months of age) are roughly symmetrical bookends in the accepted range of answers, and that early pre-natal views fall outside the standard spectrum of views. This sense may reflect views advocated in the literature at the time of the 2023 article. However, the present survey suggests a different distribution of views in the populations under discussion here. According to the survey results, 44% of respondents endorse a late pre-natal view while only 6% endorse a late post-natal view. A further 13% of respondents endorse an early pre-natal view, which lies outside Bayne et al’s spectrum of candidate answers.

Here we see that the survey results can usefully clarify sociological claims about views on infant consciousness**.** Future surveys carried out at regular intervals may also help to clarify trends in how these views change over time. They may also survey broader and more representative populations, asking more precisely formulated questions about a broader range of topics from a wider range of traditions. In this way, we may come to have a better-informed picture of views about infant consciousness among scientists, philosophers, and other populations, and a better understanding of how the field is developing over time.

## Data Availability

Anonymized data will be made available on reasonable request to the corresponding author.
